# Distribution of health anxiety in a general adult population and associations with demographic and social network characteristics

**DOI:** 10.1017/S0033291720004122

**Published:** 2022-09

**Authors:** Anja Davis Norbye, Birgit Abelsen, Olav Helge Førde, Unni Ringberg

**Affiliations:** Department of Community Medicine, UiT The Arctic University of Norway, Postbox 6050 Langnes, 9037 Tromsø, Norway

**Keywords:** Epidemiology, health anxiety, Whiteley Index

## Abstract

**Background:**

Health anxiety (HA) is associated with increased risk of disability, increased health care utilization and reduced quality of life. However, there is no consensus on which factors are important for the level of HA. The aim of this study was to explore the distribution of HA in a general adult population and to investigate whether demographic and social factors were associated with HA.

**Methods:**

This study used cross-sectional data from the seventh Tromsø study. A total of 18 064 participants aged 40 years or older were included in the analysis. The six-item Whiteley Index (WI-6) with a 5-point Likert scale was used to measure HA. Sociodemographic factors included age, sex, education, household income, quality of friendship and participation in an organized activity.

**Results:**

HA showed an exponential distribution among the participants with a median score of 2 points out of 24 points. In total, 75% had a total score of 5 points or less, whereas 1% had a score >14 points. Education, household income, quality of friendship and participation in organized activity were significantly associated with HA. The variable quality of friendship demonstrated the strongest association with HA.

**Conclusion:**

Our study showed an exponential distribution of HA in a general adult population. There was no evident cut-off point to distinguish participants with severe HA based on their WI-6 score, indicating the importance of analysing HA as a complex, continuous construct. HA demonstrated strong associations with quality of friendship and participation in an organized activity.

## Introduction

Being concerned about one's own health serves an important adaptive function – it increases survival. Dealing with symptoms of illness in a timely manner is beneficial. However, some individuals become overly health anxious and develop worries that range from mild to extreme concerns (Rachman, 2012). This is called health anxiety (HA). Severe HA has been found to increase health care use (Barsky, Ettner, Horsky, & Bates, 2001; Bobevski, Clarke, & Meadows, 2016) and the risk of long-term sick leave (Eilenberg, Frostholm, Schroder, Jensen, & Fink, 2015) and is a persistent condition if left untreated (Fink, Ornbol, & Christensen, 2010). HA is often associated with other anxiety disorders, such as panic disorder and generalized anxiety disorder (Sunderland, Newby, & Andrews, 2013). Despite growing evidence of the consequences of HA, we know little about the distribution of HA in the general population. One reason is that most research on HA has been performed with patient populations (Bilani et al., 2019; Seivewright et al., 2004; Tyrer et al., 2011).

### Measurement of HA

The three most frequently used self-report measures to assess HA, all originally developed for screening purposes for the diagnosis of hypochondriasis, are the Short Health Anxiety Inventory (SHAI) (Salkovskis, Rimes, Warwick, & Clark, 2002), the Illness Attitude Scale (IAS) (Kellner, Abbott, Winslow, & Pathak, 1987) and the Whiteley Index (WI) (Pilowsky, 1967). The WI is a self-rated instrument developed in 1967 as a diagnostic tool for hypochondriasis but later began to be used to screen for HA. The use of diverse self-report measures makes comparisons between screening studies difficult.

### Distribution of HA

There is no consensus on the appropriate cut-off points to define HA with different versions of WI, although most studies have chosen to set a cut-off point. Hedman et al. (2015) have recommended a cut-off point of 5 with a WI with 14 questions and dichotomous response options. However, no other studies have reported this threshold in population studies. The lack of criteria to define HA through the different self-report measures is a challenge within this field of research. This affects the reliability and validity of assessments of HA prevalence, which has been reported to be between 2.7% and 13%. A study based on the IAS estimated a point prevalence of 6% in a German population (Bleichhardt & Hiller, 2007), but the representativeness of the sample was dubious because most participants in the sample had low education levels. Martin and Jacobi (2006), however, found an overall 12-month prevalence of 2.75%, using diagnostic interviews for screening purposes. In New Zealand, a convenience sample of elderly individuals over 65 years found a prevalence of 7% with the SHAI (Boston & Merrick, 2010). Sunderland et al. (2013) observed a point prevalence of 3.4% and a 12-month prevalence of 4.2% based on an Australian national survey. However, their assessment was restricted by the use of only one single question: ‘Have you ever worried a lot about serious illness despite reassurance from a doctor?’ Furthermore, two articles from Canada and the USA reported a prevalence of illness worry (Looper & Kirmayer, 2001; Noyes, Carney, Hillis, Jones, & Langbehn, 2005) of 6% and 13%, respectively. These authors assessed illness worry based on the same question but asked the respondents to report based on different time periods: ‘In the past 12 months, have you had a period of 6 months (Looper & Kirmayer, 2001) or 1 month (Noyes et al., 2005) or more when you worried about having a serious physical illness most of the time?’

In line with Ferguson (2009), we assume that HA is more accurately represented as a dimensional rather than a categorical construct. To the best of our knowledge, there have been no studies exploring the distribution of HA in a general population, despite the belief that HA is experienced on a continuum ranging from mild concerns to severe anxiety (Ferguson, 2009). The Tromsø study: Tromsø 7, a health survey that included all adult inhabitants in the municipality of Tromsø, Norway, has given us a rare opportunity to describe the different degrees of HA in a general population. The aim of this article is to study the distribution of a self-report HA measure in a general adult population and the association of HA with sociodemographic variables. In the available data set, HA was measured with the six-item WI (WI-6).

## Method

### Study design and population

This study used cross-sectional, self-reported data from the Tromsø study: Tromsø 7. The Tromsø study is a large Norwegian population-based health survey that includes the collection of self-reported data, interviews, physical examinations and the collection of biological material. Seven surveys have been conducted since 1974 with different birth cohorts. The Tromsø study is described in detail elsewhere (Jacobsen, Eggen, Mathiesen, Wilsgaard, & Njolstad, 2012). The current Tromsø study, Tromsø 7, was conducted in 2015–2016. All inhabitants in the municipality of Tromsø aged 40 years or older were invited, for a total of 32 591 men and women. A questionnaire was included in the invitation, which the participants brought with them in a filled-out form to the clinical examination. At the examination, participants provided additional self-reported information on a wide range of topics, including sociodemographic and health-related information. The invited participants received one reminder if they did not attend their examination. By the end of 2016, 21 083 participants had taken part in Tromsø 7, representing an attendance rate of 65%. A total of 67% of the invited women and 62.5% of the invited men participated in the study. However, the participation rates were lower among participants 80 years or older, with an attendance rate of 34% for women and 48% for men.

### Variables

#### Dependent variable

We measured HA using the one-factor, WI-6. The WI originally consisted of 14 questions, each to be answered with a false/true response. Today, versions of the WI are available with 14, 13, 11, 10, 8, 7 and 6 items with both true/false and 5-point Likert scale response options (Welch, Carleton, & Asmundson, 2009). The 5-point Likert scale version is considered beneficial for use in a general population (Welch et al., 2009) as it is easier for respondents to use (Preston & Colman, 2000) and captures the continuity of a phenomenon (Ferguson, 2009).

Table 1 provides an overview of the WI-6 questions. All respondents answered each question with one of the following response options: ‘not at all’, ‘to some extent’, ‘moderately’, ‘to a considerable extent’ or ‘to a great extent’. Item scores were transformed into values from 0 to 4 (0 representing ‘not at all’ and 4 representing ‘to a great extent’), and the item scores were summed to a total score (*Y*) ranging from 0 to 24, where *Y* = 0 represented no HA and *Y* = 24 represented the highest possible measurement of HA. In the questionnaire, the introduction (‘In the past 12 months, have you…’) was omitted, which limited our knowledge of which time frame the participants used as a reference.

**Table 1.** Questions included in the WI-6

Question	Text
1	Do you think there is something seriously wrong with your body?
2	Do you worry a lot about your health?
3	Is it hard for you to believe the doctor when he tells you there is nothing to worry about?
4	Do you often worry about the possibility that you have a serious illness?
5	If a disease is brought to your attention (e.g. via TV, radio, internet, newspapers or someone you know), do you worry about getting it yourself?
6	Do you find that you are bothered by many different symptoms?

#### Demographic and social variables

Since the existing literature has reported inconsistent findings on the background demographic characteristics associated with HA, we chose to include both demographic and social variables. Age on 31 December 2015 was reported and was included both as a continuous variable and in 10-year age groups. As only 16 participants were over 90 years of age, the two oldest age groups were merged into ‘80 years or older’. Education was reported as ‘the highest level of education you have completed’, with four categories: primary education up to 10 years of schooling, vocational/upper secondary education (minimum 3 years), college/university (< 4 years) or college/university (≥ 4 years). The wording of the household income question was as follows: ‘What was the household's total taxable income last year?’, with eight categories ranging from ‘less than 150 000 Norwegian kroner (NOK)’ (approximately 12 000 British pound Stirling (GBP) to ‘more than 1 million NOK’ (80 000 GBP). The household income categories were merged into four categories: low (less than NOK 451 000), lower middle (NOK 451– 750 000), upper middle (NOK 751 000–1 million) or high (more than NOK 1 million). The national average value (data from Statistics Norway) was set as the reference value in the regression analysis for both the education and household income variables.

Participants were asked two questions concerning their family life: ‘Do you live with a spouse/partner?’ and ‘How many children do you have?’ Participants reported their biological children, adopted children, stepchildren and foster children. We merged these alternatives into one dichotomized variable named ‘Children’, where 0 indicated ‘no’ responses to all of the alternatives and 1 indicated a ‘yes’ response to one or more of the alternatives.

We also included two questions about the quality of friendship: ‘Do you have enough friends who can give you help and support when you need it?’ and ‘Do you have enough friends you can talk confidentially with?’ These two variables were highly correlated and were merged into one variable named ‘Close friends’. This new variable included three categories: ‘No’, for those who answered ‘no’ to both original questions; ‘To some extent’, for those who answered ‘yes’ to only one original question; and ‘Yes’, for those who answered ‘yes’ to both original questions. Finally, the participants rated their participation in the organized activity with the following options: ‘never or just a few times a year’, ‘1–2 times a month’, ‘approximately once a week’ or ‘more than once a week’.

### Statistical analysis

All analyses were performed with STATA version 15.1 (STATA Corp LP, College Station, Texas, USA). Participants with missing values for the dependent variable (HA) were excluded prior to the analyses. In the descriptive analyses, the means were calculated for continuous variables, and the frequency distributions were calculated for the categorical variables. Due to the non-normal distribution of the dependent variable (HA), we considered both a negative binomial distribution and a decreasing exponential distribution to model any association between HA (*Y*) and the relevant covariables (*X_i_*). Exponential regression gave the best fit when tested with the Akaike information criterion (AIC) and Bayes information criterion (BIC). We, therefore, used a multivariate exponential regression model in both the bivariate and multivariate analyses:
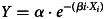
*i* *=* *1,….,n; n* = number of covariables

The covariables age and gender were included in all analyses. Only statistically significant covariables were included in the final multivariate model. The level of statistical significance (*p* value) was set at 0.05. We also conducted survey weighting analyses to adjust for non-response, which gave identical results as the unweighted analyses. The presented results are from the unweighted analyses.

The dependent variable (HA) in the estimated model is presented with the exponential beta [exp(b)], where exp(b) describes the percentage difference in the WI-6 score relative to the reference category for the different covariables with all else held constant. We found a significant interaction between age and household income and performed an additional analysis with age-stratified groups: we categorized those under 67 as ‘working-age participants’ and those 67 years or older as ‘retirement-age participants’.

### Ethics

The study was approved by the Regional Committee for Medical and Health Research Ethics (ID 2016/1793). All participants gave written informed consent before admission.

## Results

### Participant characteristics and the distribution of HA in the study population

A total of 21 083 persons between 40 and 99 years old participated in this study; 52.5% were women, and the mean age was 56 (s.d. 11) years. A total of 817 participants had one or more missing items on the WI-6 and were excluded from the analysis.

**Fig. 1.**
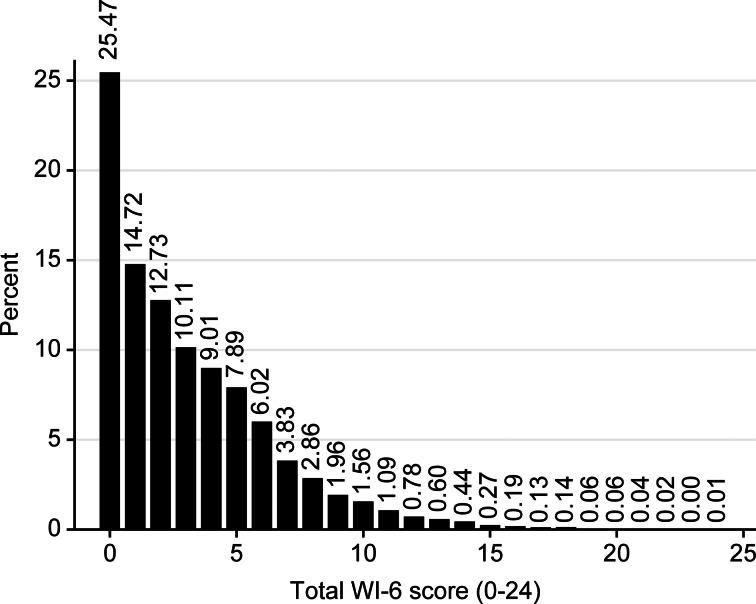
The distribution of HA in the population as measured by the WI-6. *N* = 20 266 persons.

The distribution of HA was highly skewed with exponential distribution (Fig. 1). The mean and median scores of the WI-6 were 3.3 and 2 points, respectively. In total, 75% of the participants had a total score of 5 points or less, 5% had a score above 10 points and 1% had a total score of more than 14 points. The study population's demographic and social characteristics, including the mean WI-6 scores in the participant subgroups, are listed in Table 2. The WI-6 scores ranged from 0 to 20–24 in all subgroups categorized according to the demographic and social variables.

**Table 2.** Demographic and social factors of the respondents, including the mean HA value as measured by the WI-6, by respondent characteristics

Variable				Mean HA value indicated by the WI-6	Percent with 5 points or above, WI-6	Percent with 10 points or more, WI-6
	Categories	*N*	Percent			
Age	40–49 years	6 432	31	3.2	28	6
	50–59 years	6 035	29	3.2	28	6
	60–69 years	5 179	25	3.0	27	5
	70–79 years	2 676	13	3.0	26	4
	80 years or older	761	4	3.3	27	4
	Total	21 083				
Gender	Female	11 074	53	3.1	27	5
	Male	10 009	47	3.1	27	5
	Total	21 083				
Educational level	Primary education	4 796	23	3.5	28	7
	Vocational/Upper secondary ed.	5 756	28	3.2	28	5
	College/University <4 years	4 008	19	3.2	23	5
	College/University ⩾4 years	6 145	30	2.8	27	4
	Total	20 705				
Household income	Low (less than NOK 451 000)	4 545	20	3.8	34	8
	Lower middle (NOK 451–750 000)	5 884	29	3.3	29	5
	Upper middle (NOK 751 000–1 million)	4 741	25	3.1	27	5
	High (more than NOK 1 million)	5 015	27	2.5	20	3
	Total	20 185				
Do you live with a spouse/partner?	No	4 609	22	3.5	31	7
	Yes	15 283	77	3.0	26	5
	Total	19 892				
Do you have children?	No	1 711	8	3.1	30	7
	Yes	18 705	92	3.9	27	5
	Total	20 416				
Do you have friends you can get support from and talk confidentially with? (‘Close friends’)	No	1 621	8	4.9	46	14
	To some extent	1 774	9	4.2	38	10
	Yes	17 117	83	2.9	24	4
	Total	20 512				
Organized activity	Never or just a few times a year	11 310	55	3.3	29	6
	1-2 times a month	4 981	24	3.0	26	5
	Approximately once a week	2 587	13	2.9	25	4
	More than once a week	1 856	9	2.7	23	4
	Total	20 744

### Associations between HA and sociodemographic factors

In the bivariate exponential regression analyses, all variables except gender were significantly associated with HA (results not shown). In the multivariate analysis, the variables concerning family life (‘Do you have a spouse/partner’ and ‘Do you have children’) were non-significant and were excluded from the final model. Table 3 shows the final model including the demographic and statistically significant social factors.

**Table 3.** Associations between HA, as measured by the WI-6, and relevant sociodemographic factors according to the multivariate exponential regression

Variable		Exp(b)	95% CI^a^
Age, by 10-year age groups		0.93^c^	0.92–0.95
Gender	Female	1	
	Male	1.01	0.98–1.04
Education	Primary education	1.04^b^	1.00–1.09
	Vocational/Upper secondary ed.	1	
	College/University <4 years	1.03	0.99–1.08
	College/University ⩾4 years	0.95^c^	0.91–0.98
Household income	Low (less than 451 000 NOK)	1.17^c^	1.12–1.21
	Lower middle (451–750 000 NOK)	1	
	Higher middle (751 000–1 million NOK)	0.93^c^	0.89–0.96
	High (>1 million NOK)	0.78^c^	0.75–0.81
Do you have friends you can get support from and talk confidentially with? (‘Close friends’)	Yes	1	
	To some extent	1.42^c^	1.34–1.49
	No	1.61^c^	1.53–1.70
Organized activity	Never or just a few times a year	1	
	1–2 times a month	0.97	0.94–1.01
	Once a week	0.95^b^	0.91–0.99
	More than once a week	0.88^c^	0.83–0.92
Constant		4.5	4.13–4-94

a 95% CI = 95% confidence interval.

b Significant below 0.05 level.

c Significant below 0.01 level.

*N* = 18 984 persons

Respondents who did not have friends to get support from and talk confidentially with had significantly higher HA than those who did have such friends (Table 3). Answering ‘no’ to the ‘Close friends’ questions was associated with a 61% higher WI-6 score while answering ‘to some extent’ was associated with a 41% higher WI-6 score compared to that of the reference category (answering ‘yes’). Similarly, regarding the frequency of participation in organized activity, participating in an organized activity once a week or more was associated with significantly lower HA than the other categories. Household income showed a negative association with HA. Gender was not significantly associated with HA.

We found age to be negatively associated with WI-6 scores. In addition, there was a significant interaction between age and household income. A stratified analysis for age was therefore performed, with the participants divided into working-age participants and retirement-age participants, as shown in Table 4. The mean HA value was similar in both groups. The stratified analysis showed that HA decreased with increasing age among working-age participants but showed no association with age among retirement-age participants. The quality of friendship remained strongly negatively associated with HA in both strata. Participation in organized activity was significantly negatively associated with HA among retirement-age participants who reported participating weekly, but this relationship was non-significant among working-age participants. In addition, the associations of HA with both education and household income were non-significant in the retirement-age participant group.

**Table 4.** Associations between HA, as measured by the WI-6, and relevant sociodemographic factors, stratified by age

		<67 years *N* = 15 132		≥67	*N* = 3 852
Variable		Exp(b)	^a^95% CI	Exp(b)	95% CI^a^
Age, by 10-year age groups		0.94^c^	0.92–0.96	1.00	0.94–1.07
Gender	Female	1		1	
	Male	1.01	0.98–1.05	1.00	0.93–1.06
Education	Primary education	1.04	1.00–1.09	1.10^b^	1.01–1.19
	Vocational/Upper secondary ed.	1		1	
	College/University <4 years	1.02	0.99–1.08	1.08	0.97–1.19
	College/University ⩾4 years	0.93^c^	0.91–0.98	1.06	0.96–1.18
Household income	Low (less than 451 000 NOK)	1.24^c^	1.18–1.31	1.04	0.97–1.12
	Lower middle (451–750 000 NOK)	1		1	
	Higher middle (751 000–1 million NOK)	0.93^c^	0.89–0.97	0.93	0.83–1.04
	High (>1 million NOK)	0.78c^c^	0.74–0.81	0.97	0.84–1.12
Do you have friends you can get support from and talk confidentially with? (‘Close friends’)	Yes	1		1	
	To some extent	1.41^c^	1.33–1.49	1.42^c^	1.28–1.58
	No	1.61^c^	1.52–1.71	1.60^c^	1.42–1.80
Organized activity	Never or just a few times a year	1		1	
	1–2 times a month	0.98	0.94–1.02	0.96	0.88–1.04
	Once a week	0.97	0.92–1.02	0.89^b^	0.80–0.98
	More than once a week	0.87^c^	0.82–0.92	0.89	0.79–1.00
Constant		4.29	3.79–4.85	2.63	1.67–4.14

a 95% CI = 95% confidence interval.

b Significant below 0.05 level.

c Significant below 0.01 level.

## Discussion

### Distribution of HA

In the present study, HA in the population is explored as a continuous rather than a dichotomous characteristic. We found an exponential distribution of HA on the WI-6 scale ranging from 0 to 24. There was no evident cut-off point to distinguish participants with severe HA based on their WI-6 scores, indicating the importance of analyzing HA as a complex, continuous construct. To the best of our knowledge, this is the first study to report HA as a continuous phenomenon in a general population. Although the mean HA level was low, there was great variation within all participant subpopulations. This finding illustrates the continuity of HA. We found no difference in the level or presence of HA related to gender, which is in concordance with other epidemiological studies on HA (Boston & Merrick, 2010; Martin & Jacobi, 2006; Sunderland et al., 2013). Although women are reported to have a higher prevalence of other anxiety disorders than men (Bekker & van Mens-Verhulst, 2007; Flensborg-Madsen, Tolstrup, Sorensen, & Mortensen, 2012), this does not appear to be the case with HA. In light of our results, we think it is relevant to also explore the consequences of HA-related health care use, disability and comorbidity for slight and moderate as well as severe HA.

Because we used a representative sample rather than a convenience sample, our results show a valid distribution of HA in a general adult population. In addition, by using a validated measurement tool rather than one question, we were able to better capture the complex nature of HA. Finally, most of the research on the prevalence of HA was published before 2010 (Bleichhardt & Hiller, 2007; Looper & Kirmayer, 2001; Martin & Jacobi, 2006; Noyes et al., 2005). Therefore, our research is important to provide updated knowledge of the occurrence of HA in a general population.

### The quality of friendship and participation in organized activity

To the best of our knowledge, this is the first report showing that the quality of friendship is highly associated with HA. Our results show that 17% of the population indicated that they had no or little perceived support and confidentiality from friends. Interestingly, our model estimated that compared to those who answered yes to both ‘Close friends’ questions, participants who did not report such high quality of friendship had 42–61% higher HA. As there is some overlap between the different anxiety disorders (Zimmermann, Chong, Vechiu, & Papa, 2020), it is relevant to examine the association of friendship with other anxiety disorders. One prospective study found that the quality and quantity of social networks were non-significant for the development of anxiety disorders, whereas perceived loneliness was a significant predictor of anxiety (Flensborg-Madsen et al., 2012). The questions in our study related to the quality of friendship might be interpreted similarly to questions about perceived loneliness. In our study, reduced perceived quality of friendship was the single most important factor associated with the level of HA.

Perceived loneliness may also play a part in the negative association between HA and participation in organized activity. In our study, we did not differentiate participation in physical or other social activities, as both types of activities are thought to be overall beneficial for health (Bygren, Konlaan, & Johansson, 1996; Dore, O'Loughlin, Beauchamp, Martineau, & Fournier, 2016). Participation in organized activity may increase survival (Bygren et al., 1996), and it is associated with higher life satisfaction and lower anxiety and depression (Cuypers et al., 2012). In a review paper on anxiety disorders, exercise was found to be a protective factor for the development of anxiety disorders (Zimmermann et al., 2020). This might be in concordance with our cross-sectional results, which showed that participation in an organized activity once a week or more was significantly associated with low levels of HA.

### Interaction between age and household income on HA

In the stratified analyses, we found that while household income was highly associated with the level of HA in the working-age group, this factor was not significantly associated with HA for the retirement-age group. This finding may suggest that factors other than household income are associated with HA in older participants. The difference in significant associations may also indicate differences between the two groups. Most of the participants aged 67 years or older were retired, which would have reduced the differences in their income, as most retired participants would have a percentage-wise reduction in income. We also identified descriptive differences between the two groups related to education, with higher education being more common among younger participants. However, in the retirement-age group, we also found a large variation in maximum HA, ranging from 14 to 22 points out of 24 points.

Interestingly, we found age to be negatively associated with HA up to the age of 67, indicating a decline in HA in the retirement-age groups. This association is in accordance with the results of Sunderland et al. (2013) on a population aged 15–85 years. They found that the prevalence of HA was lowest in the youngest and oldest participants, with a peak in middle age. A similar trend was observed in our study among participants 40 years and older. Interestingly, the negative association of age with HA was non-significant among retirement-age participants. The findings of Boston and Merrick (2010), who found no association between HA and age in a population over 65 years, are also in accordance with our results. We cannot conclude whether an age or cohort effect can explain this finding. If it is an age effect, we would expect similar findings in future research. If our HA research illustrates a cohort effect, we can expect an increasing number of people with HA in the years to come.

### Strength and limitations

A strength of our study is the large study sample and high participation rate compared to other population-based studies (Langhammer, Krokstad, Romundstad, Heggland, & Holmen, 2012). In addition, we chose to use the recommended 5-point Likert scale, which has been found to have better psychometric properties than dichotomous options (Welch et al., 2009). Previous studies using different versions of the WI have mostly used dichotomous response options. The WI is recommended for use as a screening tool for HA (Weck, Richtberg, & Neng, 2014) and is beneficial for use in population health surveys because of its relatively limited number of questions compared to the SHAI (14 or 18 questions) or the IAS (29 questions). We hope that the use of a recommended measurement tool for a general population will motivate other studies to include this relatively short measurement of HA in future health surveys. More studies are needed to further explore the usefulness of this procedure.

Despite the representative sample in our study, a Norwegian study found that people with mental illness were less likely than those without mental illness to participate in population surveys (Langhammer et al., 2012). However, the Tromsø study is marketed as a health check in addition to a health survey. In contrast to other anxiety disorders, HA is characterized by the seeking of reassurance for bodily stress and fears (Mykletun et al., 2009). We therefore do not believe that HA-related avoidance has affected participation in the Tromsø study.

In the questionnaire, the introduction (‘In the past 12 months, have you…’) was omitted. Unfortunately, this limited our knowledge of which time frame the participants used as a reference. There is a lack of knowledge of the variation in low and moderate HA over time. However, severe HA is persistently present, with little variation over time periods (Fink et al., 2010).

When the participants gave self-reported information regarding HA, they answered the seventh question: ‘Do you have recurring thoughts about having a disease that is difficult to be rid of?’ We decided to not include this question in our study in line with the recommended use of the WI-6 (Veddegjaerde, Sivertsen, Wilhelmsen, & Skogen, 2014). This seventh question was not validated for use in a general population. All analyses with both versions of the questionnaire gave identical results. Therefore, we do not believe that excluding the seventh question influenced our results.

## Conclusion

In conclusion, our study demonstrates the continuity that is believed to characterize HA. Future studies should explore the impacts of this continuum. The findings indicate that social factors of friendship and participation in an organized activity may be decisive for HA levels.
